# Supplementary material for: Modelling Skylarks (Alauda arvensis) to Predict Impacts of Changes in Land Management and Policy: Development and Testing of an Agent-Based Model
Source: PLoS One. 2013 Jun 6;8(6):e65803. doi: 10.1371/journal.pone.0065803 (PMC3675089; doi:10.1371/journal.pone.0065803)
Supplement: Supporting Information S4 — The skylark ODdox as a zipped archive. (ZIP) [file pone.0065803.s004.zip › Skylark_ODdox/functions_vars_0x70.html]

ALMaSS Skylark ODdox: Class Members - Variables


ALMaSS Skylark ODdox
 2.0


- Main Page
- Related Pages
- Classes
- Files

- Class List
- Class Index
- Class Hierarchy
- Class Members

- All
- Functions
- Variables
- Typedefs

- a
- b
- c
- d
- e
- f
- g
- h
- i
- l
- m
- n
- o
- p
- r
- s
- t
- v
- x
- y

### - p -

- Paired
  : Skylark\_Adult
- Pct
  : PermCropData
- pct
  : tpct
- Percent
  : Rotation
  , Starter
- PolyHeightData
  : SkTerritories
- PolyRefData
  : SkTerritories
- PolySizeData
  : SkTerritories
- PolyTypeData
  : SkTerritories
- ProbesSet
  : Population\_Manager


- Generated on Thu Jan 10 2013 13:15:39 for ALMaSS Skylark ODdox by
   1.8.1.1
